# Supplementary material for: Insulin therapy adherence and its associated factors among diabetic patients in a Ghanaian primary care hospital
Source: PLoS One. 2025 Jan 24;20(1):e0312094. doi: 10.1371/journal.pone.0312094 (PMC11760006; doi:10.1371/journal.pone.0312094)
Supplement: Attachment — Submitted filename: Reviewed - PONE-D-24-23124.pdf [file pone.0312094.s001.pdf]

PLOS ONE

Insulin Therapy Adherence and its Associated Factors among Diabetic Patients in a Ghanaian Primary Care Hospital

--Manuscript Draft--

**Manuscript Number:**	PONE-D-24-23124
**Article Type:**	Research Article
**Full Title:**	Insulin Therapy Adherence and its Associated Factors among Diabetic Patients in a Ghanaian Primary Care Hospital
**Short Title:**	Assessment of Insulin Therapy Adherence
**Corresponding Author:**	Israel Abebrese Sefah, MPH<br>University of Health and Allied Sciences<br>Ho, GHANA
**Keywords:**	Keywords: Adherence, insulin, diabetes mellitus, Medication Adherence Reporting Scale-5, Ghana
**Abstract:**	Background<br>Diabetes mellitus (DM) is a global health problem. Adherence to intensive insulin therapy is necessary to achieve better glycemic control in all types of diabetes. This study aimed to evaluate the extent of adherence to insulin therapy, its predictors and to identify barriers to its adherence.<br><br>Method<br>This was a cross-sectional survey among adult (≥18 years) diabetic patients who are currently using insulin, either alone or in combination with an oral antidiabetic regimen, and seeking primary care at Kwame University of Science and Technology Hospital in Ghana. A total of one hundred and eight-six patients were conveniently sampled, and interviewed. Insulin adherence was determined using the Medication Adherence Reporting Scale-5. Descriptive statistics, chi square test of independence and a multiple logistic regression analysis were performed using STATA version 14 (StataCorp, TX USA).<br><br>Results<br>The majority of the patients interviewed were over 60 years (40.32%); female (61.83%); married (68.82%); and had completed secondary education (48.39%). 67.20% of the patients were adherent to insulin therapy. Adherence level was associated with age (p=0.020), marital status (p=0.001), employment status (p=0.012), type of DM (p<0.001), regular follow-up (p=0.007) and comorbidities (p=0.002) and was only predicted by the type of DM (aOR=14.82 C.I 1.34 – 163.50, p-value = 0.028).<br><br>Conclusion<br>Adherence to insulin therapy among our study population was suboptimal, which is a concern considering the associated increased risk of complications. Adherence assessment and counselling by healthcare professionals to address barriers to poor adherence must be continually undertaken to achieve optimal glycemic control.
**Order of Authors:**	Israel Abebrese Sefah, MPH
	Michael Mensah, PharmD
	Araba Ata Hutton-Nyameaye, PhD
	Emmanuel Sarkodie, PharmD
	Johanna C Meyer, PhD
	Brian Godman, PhD
	Varsha Bangalee, PhD
**Additional Information:**	
**Question**	**Response**

Powered by Editorial Manager® and ProduXion Manager® from Aries Systems Corporation

**Financial Disclosure**<br><br>Enter a financial disclosure statement that describes the sources of funding for the work included in this submission. Review the submission guidelines for detailed requirements. View published research articles from *PLOS ONE* for specific examples.<br><br>This statement is required for submission and **will appear in the published article** if the submission is accepted. Please make sure it is accurate.<br><br>**Funded studies**<br>Enter a statement with the following details:<br>• Initials of the authors who received each award<br>• Grant numbers awarded to each author<br>• The full name of each funder<br>• URL of each funder website<br>• Did the sponsors or funders play any role in the study design, data collection and analysis, decision to publish, or preparation of the manuscript?<br><br>Did you receive funding for this work?	The author(s) received no specific funding for this work.
**Competing Interests**<br><br>Use the instructions below to enter a competing interest statement for this submission. On behalf of all authors, disclose any competing interests that could be perceived to bias this work—acknowledging all financial support and any other relevant financial or non-financial competing interests.<br><br>This statement is required for submission and **will appear in the published article** if the submission is accepted. Please make sure it is accurate and that any funding sources listed in your Funding Information later in the submission form are also declared in your Financial Disclosure statement.	The authors have declared no competing interests exist

View published research articles from *PLOS ONE* for specific examples.<br><br>**NO authors have competing interests**<br><br>Enter: *The authors have declared that no competing interests exist.*<br><br>**Authors with competing interests**<br><br>Enter competing interest details beginning with this statement:<br><br>*I have read the journal's policy and the authors of this manuscript have the following competing interests: [insert competing interests here]*<br><br>* typeset	
**Ethics Statement**<br><br>Enter an ethics statement for this submission. This statement is required if the study involved:<br><br>• Human participants<br>• Human specimens or tissue<br>• Vertebrate animals or cephalopods<br>• Vertebrate embryos or tissues<br>• Field research<br><br>Write "N/A" if the submission does not require an ethics statement.<br><br>General guidance is provided below. Consult the submission guidelines for detailed instructions. **Make sure that all information entered here is included in the Methods section of the manuscript.**	Ethical approval was secured from the University of Health and Allied Sciences' Research Ethics Committee (UHAS-REC A.7 [39] 22-23) and administrative approval was obtained from management of the KNUST Hospital. Written informed consent was secured from each study participant before the interview and personal identifiers were excluded during the data collection to safeguard confidentiality.

Powered by Editorial Manager® and ProduXion Manager® from Aries Systems Corporation

**Format for specific study types**<br><br>**Human Subject Research (involving human participants and/or tissue)**<br>• Give the name of the institutional review board or ethics committee that approved the study<br>• Include the approval number and/or a statement indicating approval of this research<br>• Indicate the form of consent obtained (written/oral) or the reason that consent was not obtained (e.g. the data were analyzed anonymously)<br><br>**Animal Research (involving vertebrate animals, embryos or tissues)**<br>• Provide the name of the Institutional Animal Care and Use Committee (IACUC) or other relevant ethics board that reviewed the study protocol, and indicate whether they approved this research or granted a formal waiver of ethical approval<br>• Include an approval number if one was obtained<br>• If the study involved *non-human primates*, add *additional details* about animal welfare and steps taken to ameliorate suffering<br>• If anesthesia, euthanasia, or any kind of animal sacrifice is part of the study, include briefly which substances and/or methods were applied<br><br>**Field Research**<br><br>Include the following details if this study involves the collection of plant, animal, or other materials from a natural setting:<br>• Field permit number<br>• Name of the institution or relevant body that granted permission	
**Data Availability**<br><br>Authors are required to make all data underlying the findings described fully available, without restriction, and from the time of publication. PLOS allows rare exceptions to address legal and ethical concerns. See the PLOS Data Policy and FAQ for detailed information.	Yes - all data are fully available without restriction

A Data Availability Statement describing where the data can be found is required at submission. Your answers to this question constitute the Data Availability Statement and **will be published in the article**, if accepted.<br><br>**Important:** Stating 'data available on request from the author' is not sufficient. If your data are only available upon request, select 'No' for the first question and explain your exceptional situation in the text box.<br><br>Do the authors confirm that all data underlying the findings described in their manuscript are fully available without restriction?	
**Describe where the data may be found in full sentences. If you are copying our sample text, replace any instances of XXX with the appropriate details.**<br><br>• If the data are **held or will be held in a public repository**, include URLs, accession numbers or DOIs. If this information will only be available after acceptance, indicate this by ticking the box below. For example: *All XXX files are available from the XXX database (accession number(s) XXX, XXX.*).<br>• If the data are all contained **within the manuscript and/or Supporting Information files**, enter the following: *All relevant data are within the manuscript and its Supporting Information files.*<br>• If neither of these applies but you are able to provide **details of access elsewhere**, with or without limitations, please do so. For example:<br><br>*Data cannot be shared publicly because of [XXX]. Data are available from the XXX Institutional Data Access / Ethics Committee (contact via XXX) for researchers who meet the criteria for access to confidential data.*<br><br>*The data underlying the results presented in the study are available from (include the name of the third party*	All relevant data are within the manuscript

and contact information or URL).<br>• This text is appropriate if the data are owned by a third party and authors do not have permission to share the data.<br><br>* typeset	
Additional data availability information:	

Powered by Editorial Manager® and ProduXion Manager® from Aries Systems Corporation

Manuscript

Click here to access/download;Manuscript;Manuscript-Without Figure.docx

Insulin Therapy Adherence and its Associated Factors among Diabetic Patients in a Ghanaian Primary Care Hospital

Assessment of Insulin Therapy Adherence

Israel Abebrese Sefah[1*,2], Michael Mensah[1], Araba Ata Hutton-Nyameaye[1], Emmanuel Sarkodie[3], Johanna C Meyer[4], Brian Godman[4,5], Varsha Bangalee[2]

1 University of Health and Allied Sciences, Volta Region, Ho PMB 31, Ghana. Email: 2017mmensah@uhas.edu.gh (MM), aharaba@uhas.edu.gh (AAHN)

2 Discipline of Pharmaceutical Sciences, School of Health Sciences, University of KwaZulu-Natal, Durban, South Africa Email: isefah@uhas.edu.gh (IAS), bangalee@ukzn.ac.za (VB)

3 University Hospital, Kwame Nkrumah University of Science and Technology, Kumasi PMB UPO KNUST, Ghana Email : esarkodie.pharm@gmail.com (ES)

4 Department of Public Health Pharmacy and Management, Sefako Makgatho Health Sciences University, South Africa. Email : brian.godman@smu.ac.za (BG); hannelie.meyer@smu.ac.za (JCM)

5 Strathclyde Institute of Pharmacy and Biomedical Science (SIPBS), University of Strathclyde, Glasgow G4 0RE, UK

* Correspondence: isefah@uhas.edu.gh

ORCID ID

Israel Abebrese Sefah – 0000-0001-6963-0519

Johanna C Meyer – 0000-0003-2557-4425

Brian Godman - 0000-0001-6539-6972

Varsha Bangalee - 0000-0002-9613-1501

Background

Diabetes mellitus (DM) is a global health problem. Adherence to intensive insulin therapy is necessary to achieve better glycemic control in all types of diabetes. This study aimed to evaluate the extent of adherence to insulin therapy, its predictors and to identify barriers to its adherence.

Method

This was a cross-sectional survey among adult (≥18 years) diabetic patients who are currently using insulin, either alone or in combination with an oral antidiabetic regimen, and seeking primary care at Kwame University of Science and Technology Hospital in Ghana. A total of one hundred and eight-six patients were conveniently sampled, and interviewed. Insulin adherence was determined using the Medication Adherence Reporting Scale-5. Descriptive statistics, chi square test of independence and a multiple logistic regression analysis were performed using STATA version 14 (StataCorp, TX USA).

Results

The majority of the patients interviewed were over 60 years (40.32%); female (61.83%); married (68.82%); and had completed secondary education (48.39%). 67.20% of the patients were adherent to insulin therapy. Adherence level was associated with age (p=0.020), marital status (p=0.001), employment status (p=0.012), type of DM (p<0.001), regular follow-up (p=0.007) and comorbidities (p=0.002) and was only predicted by the type of DM (aOR=14.82 C.I 1.34 – 163.50, p-value = 0.028).

Conclusion

Adherence to insulin therapy among our study population was suboptimal, which is a concern considering the associated increased risk of complications. Adherence assessment and counselling by healthcare professionals to address barriers to poor adherence must be continually undertaken to achieve optimal glycemic control.

Keywords: Adherence, insulin, diabetes mellitus, Medication Adherence Reporting Scale-5, Ghana

Impact of findings on practice statements

- Continuous adherence assessment and counselling must be offered to all diabetes mellitus patients on insulin therapy as part of their ambulatory care to help improve outcomes.
- Using the Medication Adherence Reporting Scale-5 to determine patient adherence levels is an easy-to-use and an inexpensive method; however, it should be used with caution due to the potential for misclassification.
- Efforts must be made to provide appropriate strategies to deal with barriers to insulin adherence at ambulatory care clinics as part of the individualized comprehensive diabetic care to reduce diabetic complications.

Introduction

Diabetes is currently one of the leading causes of death and disability globally (1-4). It was estimated that 537 million people globally had diabetes in 2021 with associated health expenditures of US$966, which is forecast to be over $1054 billion globally by 2045 if not addressed (2, 5). The increased costs are driven by increasing prevalence rates, projected to rise by 45% to 783 million adults, i.e. 1 in 8 adults, by 2045 (6). Diabetes is a particular concern in low- and middle-income countries (LMICs), which currently accounts for over 75% of the global diabetes population. (3, 6). This may be due to changing lifestyles with reduced physical activity and more sedentary habits along with cultural habits and increasing urbanization (7, 8). Overall, due to challenges with human resources and financial challenges to procure medicines and equipment, diabetes has a greater impact on morbidity and mortality in sub-Saharan Africa than any other region globally (9).

Currently in Ghana there are approximately 2.4 million people living with diabetes (10). Whilst the reported prevalence of diabetes at the national level in Ghana have ranged between 2.80% to 3.95% of the population, higher prevalence rates have been reported in different parts of Ghana and different populations (11-14). At sub-national levels, higher prevalence rates of diabetes have been reported in some regions (15). For instance, a prevalence rate of 25.2% has been reported in the Ashanti region, which is one of the 18 administrative regions in Ghana (16).

Whilst the vast majority of patients with diabetes have type 2 diabetes whose treatment may include insulin, there are an appreciable proportion of patients with type 1 diabetes in Ghana whose treatment is based solely on insulin therapy (17). However, there are issues of availability and affordability as well as the monitoring equipment among many patients in LMICs, including those in Ghana, impacting on their utilisation (17-19). This is important since achieving optimal glycaemic control using insulin has been associated with a reduction in complications in both type 1 and type 2 diabetes and a reduction in all-cause mortality (20-24).

Despite extensive research and various interventions designed to improve adherence to treatment of chronic conditions, medication non-adherence still persists among patients with diabetes, including those prescribed insulin, leading to poor health outcomes (1, 23-26). Non-adherence is attributed to several complex intertwined factors (24, 27). These include the complexity of the medication regimens, the social stigma from injecting in public, weight gain, fear of side-effects, their costs especially if high co-payments alongside economic difficulties, inconsistent hospital visits, health literacy and individual health beliefs regarding medication use (17, 20, 24, 26, 28-31).

Currently, standard insulin, including soluble insulin, insulin isophane (NPH) or premixed insulins (30/70), are listed in the Ghana Essential Medicines List (EML) and reimbursed by the National Health Insurance Scheme (NHIS). Long-acting insulin analogues are currently listed in the EML though not reimbursed by the NHIS affecting access among many poor patients (17, 32-34). As a result, combined with increasing prevalence of diabetes, there has been increasing use of NPH, premixed insulin and other listed insulins in Ghana in recent years, with very limited use of long-acting insulin analogues due their high out-of-pocket payment, whose use promotes adherence due to reduce frequency of dosing (35). There are also concerns that glucometer test strips to monitor HbA1c levels and insulin syringes and needles are currently not reimbursed by NHIS (17, 36). As a result, this may negatively impact on

adherence to insulin therapy among patients with diabetes in Ghana in practice, which may be exacerbated if patients are not active members of NHIS (37). Typically, in Ghana and across LMICs, diabetes care teams consist of different healthcare professionals who work together to provide comprehensive care to patients with diabetes at ambulatory clinics. Several studies have shown that pharmacists can help enhance adherence to the use of insulin in clinics by providing adherence counselling and regular follow up, which adherence potentially enhanced by the use of smartphones (38-40).

There is currently a paucity of research conducted in Ghana to assess adherence to insulin therapy among diabetes patients, with studies to date skewed towards assessing adherence to oral anti-diabetes medicines (30, 41-45). Consequently, there is a need to address this information gap in patients prescribed insulin in Ghana given rising rates of patients requiring insulin in Ghana and the associated costs to NHIS and others. As a result, this study was designed to evaluate the extent of adherence to insulin therapy among patients with diabetes attending ambulatory care, along with identifying key barriers to adherence to insulin therapy. It is envisaged that the findings will not only be of interest to key stakeholders in Ghana but also to other LMICs faced with similar challenges, with a growing prevalence of patients with diabetes requiring insulin.

Method

Study design and setting

A prospective hospital-based cross-sectional survey was conducted among diabetic patients who are currently using insulin as either the only treatment or part of their diabetic regimen, and attending Kwame University of Science and Technology (KNUST) Hospital in Ghana. KNUST Hospital is a government-owned primary care health facility located in the south-east part of Kumasi metropolis in the Ashanti Region of Ghana. The hospital provides medical, surgical, pharmaceutical, diagnostic and specialized services such as diabetes care and asthma care to staff and students at the university as well, as people living in the surrounding community of the university.

Target population

The study was conducted among adults (18 years and above) attending the ambulatory care diabetic clinic of KNUST. This was largely composed of staff, university students and attendants of the clinic living in communities surrounding the university.

Sample size and sampling technique

A sample size of 186 was calculated using the Raosoft Inc. online calculator (http://www.raosoft.com/samplesize.html (accessed on the 15th May, 2023), assuming an adherence level of 50% (23) and an average monthly outpatient attendance of 300, at 90% power and 95% confidence interval and additional10% increase to account for possible non-response or incomplete data. A convenience sampling method was employed in the recruitment of ambulatory diabetes patients on insulin therapy and seeking care at in the primary care facility in KNUST hospital. Inclusion criteria were adult type 1 and type 2 diabetic patients using insulin as part of their diabetes treatment regimen for at least 6 months. Diabetic patients who were not on insulin, newly diagnosed and hospitalized diabetes patients managed on insulin therapy at the time of the survey, were excluded from this study.

Data collection

Several methods have been developed previously for the assessment of medication adherence among patients with non-communicable disease, including pill counts, electronic monitoring devices and self-reported methods (46). The latter method is seen as inexpensive, easy to use and practical as it identifies the concerns contributing to non-adherence compared to the 'gold standard' method of direct observation, which has been shown to be impractical, intrusive and resource intensive (47). The Medication Adherence Reporting Scale (MARS-5) is an example of a reliable self-reported adherence monitoring instrument which has been validated and used for many chronic conditions including diabetes and hypertension (48, 49).

A structured interviewer administered questionnaire was adapted from data collection tools that we have used in similar previous studies [29 – 35]. Questionnaires were administered to study participants by two trained pharmacists (IAS & MM) at the diabetes ambulatory clinic at KNUST hospital from 1st June 2023 to 31st August 2023. Other relevant patient data were extracted from patients' medical records. The questionnaire collected socio-demographic characteristics including age, gender and payment type (NHIS or 100% cash payment) as this is a known barrier of accessibility; patient clinical details including the type of DM, duration of DM from the date of diagnosis, duration of insulin therapy, name and type of insulin and documented comorbidities. Another section of the questionnaire determined insulin adherence using the MARS-5 (49). The MARS-5 measures a patient's medication adherence using five (5) questions that assess patients' negative medication taking behavior including forgetfulness, independently varying dosages, skipping doses, quitting use of the drug and taking fewer medicines than instructed. The adherence assessment was conducted by two clinical pharmacists (IAS, MM). The assessment was undertaken using a 5-point Likert scale (1—always, 2—often, 3—sometimes, 4—rarely, and 5—never) (50, 51). Responses were summed to a total score of 25 and patients with a score of ≥ 21 were classified as adherent and those with a score of <21 as non-adherent.

Data ana[illegible]s

The collected data were checked manually for completeness and accuracy. Data were subsequently entered onto Microsoft Excel® version 2013 and imported into STAT version 14 (StataCorp, TX USA) for analysis. Descriptive statistics were generated to summarize patient socio-demographic and clinical data. A chi square test of independence was used to determine the association between independent variables collected and the study outcome (adherence versus non-adherence assessed using the MARS-5 scale). A multiple logistic regression analysis was subsequently utilized to identify independent predictors of adherence status by estimating the adjusted odd ratios at a significance level of 95% for only variable with chi-square p-values ≤0.05.

Ethical Approval

Ethical approval was secured from the University of Health and Allied Sciences' Research Ethics Committee (UHAS-REC A.7 [39] 22-23) and administrative approval was obtained from management of the KNUST Hospital. Written

informed consent was secured from each study participant before the interview and personal identifiers were excluded during the data collection to safeguard confidentiality.

Results

Socio-demographic characteristics of participants

A total of 186 patients participated in the study with a response rate of 100%. The majority of participants were over 60 years old (40.32%), followed by the 50-60 age group (24.73%); were female (61.83%) and married (68.82%). An appreciable number of participants had completed secondary education (48.39%) and tertiary education (32.26%). A third (36.56%) of the participants were unemployed and 30.11% self-employed. The most common mode of payment for insulin was "cash and carry", i.e. 100% co-payment (55.91%), followed by "insurance" (30.11%). See Table 1 for further details.

Table 1: Socio-demographic characteristics of study participants (n=186)

**Variables**	**Frequency (n)**
Age (in years)	
16-20	10 (5.38%)
21-30	13 (6.99%)
31-40	14 (7.53%)
41-50	28 (15.05%)
50-60	46 (24.73%)
>60	75 (40.32%)
Sex	
Female	115 (61.83%)
Male	71 (38.17%)
Marital status	
Married	128 (68.82%)
Divorced	10 (5.38%)
Single	23 (12.37%)
Widowed	25 (13.44%)
Highest educational level	
No formal education	27 (14.52%)
Primary education	9 (4.84%)
Secondary education	90 (48.39%)
Tertiary education	60 (32.26%)
Employment status	
Employed	32 (17.20%)
Retired	30 (16.13%)
Self-employed	56 (30.11%)
Unemployed	68 (36.56%)
Payment method	
Cash and carry	104 (55.91%)
Cash and carry +insurance	26 (13.98%)
Insurance	56 (30.11%)

Clinical characteristics of participants

The majority of the participants (85.48%) had type 2 diabetes. Only 43.21% of the participants had a controlled serum glucose (4-7mmol/L). Just under a third of patients (28.49%) had been diagnosed for between 1-5 years, with just under two thirds taking insulin for less than five years (62.90%). Mixtard 30/70 (Premixed insulin of 30% soluble and 70% isophane insulin) was the predominant type of insulin used, accounting for 91.94% of the cases. Most of the participants (76.88%) injected insulin twice daily, and most(83.87%) were taking oral antidiabetic medications in combination with their insulin therapy. Metformin (75.33%) was the most commonly used oral antidiabetic medication. The majority of participants (62.70%) had a glucometer at home for self-monitoring of their insulin. Most participants (90.32%) had regular follow-up appointments, with the majority (75.68%) receiving diabetic education. Most participants (70.81%) had comorbidities, with "hypertension" being the most prevalent (92.48%).

The overall rate of adherence to insulin therapy was 67.20%. Participants reported various barriers to insulin use including "cost" (34.5%), “side effects” (23.3%) and a combination of “cost and side effects” (23.3%). (Table 2).

Table 2: Clinical characteristics of participants

**Variables**	**Frequency (n)**
Type of Diabetes mellitus (DM) (n=186)	
Type 1	27 (14.52%)
Type 2	159 (85.48%)
Duration of diagnosis (years) (n=186)	
1-5	53 (28.49**%)**
6-10	48 (25.81%)
11-15	32 (17.20%)
16-20	26 (13.98%)
>21	27 (14.52%)
Duration of insulin therapy (years) (n=186)	
1-5	117 (62.90%)
6-10	40 (21.51%)
11-15	18 (9.68%)
16-20	7 (3.76%)
>21	4 (2.15%)
Name(s) of insulin (n=186)	
Degludec (Long acting insulin analogue)	2 (1.08%)
Isophane	10 (5.37%)
Lantus (Insulin Glargine)	3 (1.61%)
Premixed (30% soluble and 70% isophane)	171 (91.94%)
Frequency of insulin self-injection (n=186)	
Once daily	43 (23.12%)
Twice daily	143 (76.88%)
Oral antidiabetic (n=186)	
Yes	156 (83.87%)
No	30 (16.13%)
Type of oral antidiabetics (n=156)	

Metformin	116 (75.33%)
Metformin and gliclazide	6 (3.90%)
Metformin and pioglitazone	17 (11.04%)
Metformin and vildagliptin	8 (5.19%)
Others	7 (4.54%)
Ownership of glucometer (n=186)	
Yes	116 (62.70%)
No	69 (37.30%)
Regular follow up (n=186)	
Yes	168 (90.32%)
No	18 (9.68%)
Diabetic education (n=185)	
Yes	140 (75.68%)
No	45 (24.32%)
Comorbidities (n=186)	
Yes	131 (70.81%)
No	54 (29.19%)
Type of comorbidity (n=131)	
Hypertension	123 (92.48%)
Hypertension and heart failure	2 (1.50%)
Hypertension and Benign prostate hyperplasia	2 (1.50%)
Others	6 (4.52%)
Herbal medicine user (n=185)	
Yes	4 (2.17%)
No	180 (97.83%)
Fasting blood sugar (mmol/L) (n=162)	
4 – 7	70 (43.21%)
>7	92 (56.79%)
Level of Adherence (n=186)	
Adherent	125 (67.20%)
Non-adherent	61 (32.80%)

(Insert Figure 1)

Association between insulin therapy adherence and patients' characteristics

Adherence to insulin therapy was statistically associated with age (p=0.020), marital status (p=0.001), employment status (p=0.012), type of DM (p<0.001), taking oral antidiabetic medication (p=0.002), regular follow-up (p=0.007) and comorbidities (p=0.002) (Table 3).

Table 3: Bivariate analyses of factors associated with adherence to insulin therapy among participants (n=186)

**Variables**	**Total, n (%)**	**Level of Adherence**		**p-value**
		**Adherent**	**Non-adherent**	

Age category (years)				
16-20	10 (5.38%)	3 (30%)	7 (70%)	**0.020***
21-30	13 (6.99%)	5 (38.46%)	8 (61.54%)	
31-40	14 (7.53%)	9 (64.29%)	5 (35.71%)	
41-50	28 (15.05%)	20 (71.43%)	8 (28.57%)	
50-60	46 (24.73%)	32 (69.57%)	14 (30.43%)	
>60	75 (40.32%)	56 (74.67%)	19 (25.33%)	
Gender				
Female	115 (61.83%)	79 (68.7%)	36 (31.3%)	0.581
Male	71 (38.17%)	46 (64.79%)	25 (35.21%)	
Marital status				
Married	128 (68.82%)	92 (71.88%)	36 (28.13%)	**0.001***
Divorced	10 (5.38%)	7 (70%)	3 (30%)	
Single	23 (12.37%)	7 (30.43%)	16 (69.57%)	
Widowed	25 (13.44%)	19 (76%)	6 (24%)	
Highest educational level				
No formal education	27 (14.52%)	21 (77.78%)	6 (22.22%)	0.617
Primary education	9 (4.84%)	6 (66.67%)	3 (33.33%)	
Secondary education	90 (48.39%)	60 (66.67%)	30(33.33%)	
Tertiary education	60 (32.26%)	38 (63.33%)	22 (36.67%)	
Employment status				
Employed	32 (17.20%)	20 (62.5%)	12 (37.5%)	**0.012***
Retired	30 (16.3%)	24 (80%)	6 (20%)	
Self-employed	56 (30.11%)	44 (78.57%)	12 (21.42%)	
Unemployed	68 (36.56%)	37 (54.41%)	31 (45.59%)	
Religious status				
Christian	167 (89.78%)	110 (65.87%)	57 (34.13%)	0.250
Muslim	19 (10.22%)	15 (78.95%)	4 (21.05%)	
Type of DM				
Type 1	27 (14.52%)	8 (29.63%)	19 (70.37%)	**<0.001***
Type 2	159 (85.48%)	117 (73.58%)	42 (26.42%)	
Duration of DM diagnosis (years)				
1-5	53 (28.49%)	30 (56.60%)	23 (43.40%)	0.344
6-10	48 (25.81%)	34 (70.833%)	14 (29.17%)	
11-15	32 (17.20%)	24 (75%)	8 (25%)	
16-20	26 (13.98%)	17 (65.38%)	9 (34.62%)	
>21	27 (14.52%)	20 (74.07%)	7 (25.93%)	
Duration of insulin therapy (years)				
1-5	117 (62.90%)	82 (70.09%)	35 (29.9%)	0.790
6-10	40 (21.51%)	25 (62.50%)	15 (37.50%)	
11-15	18 (9.68%)	12 (66.67%)	6 (33.33%)	
16-20	7 (3.76%)	4 (57.14%)	3 (42.86%)	
>21	4 (2.15%)	2 (50%)	2 (50%)	
Mode of payment for insulin				
Cash and carry	104 (55.91%)	71 (68.27%)	33 (3.73%)	0.939
Cash and carry; insurance	26 (13.98%)	17 (65.38%)	9 (34.62%)	
Insurance	56 (30.11%)	37 (66.07%)	19 (33.93%)	
Frequency of insulin self-injection				
Once daily	43 (23.12%)	29 (67.44%)	14 (32.56%)	0.970
Twice daily	143 (76.88%)	96 (67.13%)	47 (32.87%)	

Oral antidiabetic				
Yes	156 (83.87%)	112 (71.79%)	44 (28.21%)	**0.002***
No	30 (16.13%)	13 (43.33%)	17 (56.67%)	
Having a glucometer at home				
Yes	116 (62.70%)	81 (69.83%)	35 (30.17%)	0.293
No	69 (37.30%)	43 (62.32%)	26 (37.68%)	
Regular follow up				
Yes	168 (90.32%)	118 (70.24%)	50 (29.76%)	**0.007***
No	18 (9.68%)	7 (38.89%)	11 (611.1%)	
Ever had diabetic education				
Yes	140 (75.68%)	93 (66.43%)	47 (33.57%)	0.559
No	45 (24.32%)	32 (71.11%)	13 (28.89%)	
Comorbidities				
Yes	131 (70.81%)	97 (74.05%)	34 (25.95%)	**0.002***
No	54 (29.19%)	27 (50%)	27 (50%)	
Take herbal medication				
Yes	4 (2.17%)	2 (50%)	2 (50%)	0.453
No	180 (97.83%)	122 (67.78%)	58 (32.22%)	
Fasting blood sugar (mmol/L)				
4 – 7	70 (43.21%)	52 (74.29%)	18 (25.71%)	0.168
>7	92 (56.79%)	59 (64.13%)	33 (35.87%)	

* = Significant at P-value <0.05

Independent predictors of adherence to insulin therapy

Patients' adherence to insulin therapy was independently predicted by the type of DM, where patients with type 2 DM were about 15 times more likely to adhere compared to type 1 DM patients (aOR=14.82 C.I 1.34 – 163.50, p-value = 0.028), holding the age, marital status, employment status, concomitant use of oral anti-diabetes, presence of co-morbidities and whether patients regularly visited the clinic constant (Table 4).

Table 4: Logistic regression of independent variables which showed statistically significant association and medication adherence status

**Independent variables**	**aOR**	**95% CI**	**p-value**
Age category (years)			
16-20®	1.0		
21-30	0.73	0.10 – 5.32	0.753
31-40	0.41	0.02 – 8.57	0.562
41-50	0.26	0.01 – 6.04	0.398
50-60	0.22	0.01 – 5.34	0.352
>60	0.23	0.01 – 5.64	0.369
Marital status			
Married®	1.0		
Divorced	1.20	0.27 – 5.40	0.811
Single	0.28	0.02 – 3.30	0.315
Widowed	1.84	0.60 – 5.72	0.290
Employment status			

Employed®	1.0		
Retired	2.55	0.62 – 10.42	0.193
Self-employed	2.31	0.82 – 6.50	0.112
Unemployed	1.21	0.41 – 3.61	0.729
Type of DM			
Type 1®	1.0		
Type 2	14.82	1.34 – 163.50	**0.028***
Use of oral antidiabetic			
Yes	0.19	0.02 – 2.19	0.183
No®	1.0		
Regular visit to a diabetes clinic			
Yes	2.18	0.63 – 7.62	0.221
No®	1.0		
Presence of Comorbidities			
Yes	1.73	0.65 – 4.58	0.270
No®	1.0		

* = Significant at P-value <0.05; aOR = Adjusted Odds Ratio; CI = Confidence Interval; ® = reference variable

Discussion

Previous studies have demonstrated that good adherence to insulin therapy is an essential requirement for achieving adequate glycemic control and subsequently slowing the progression of microvascular and macrovascular complications, which are mostly associated with DM [2, 28]. The level of adherence to insulin therapy seen in this study was 67.20%, which is seen as suboptimal given the high and growing burden of diabetes in Ghana alongside the growing use of insulin. This is similar to other studies conducted especially in LMICs [60, 61]. Higher adherence rates have been recorded in several other studies elsewhere [62, 63]. Most of the DM patients were taking premixed insulin, with costs lower compared to the longer acting analogs but have higher incidence of hypoglycemia compared to the latter (52, 53). The high risk of hypoglycemia may have contributed among other factors as a barrier to adherence since fear of side effects were cited by patients as a common barrier to adherence. The other important barrier reported by patients was cost as over 50% of them paid for their insulin therapy out of pocket. These barriers must be addressed as part of measures to improve glycemic control among these patients.

The level of adherence level was found to be associated with patients' age (p=0.020) with a higher proportion of patients below 31 years exhibiting non-adherence to their insulin therapy. This is consistent with other studies where busy lifestyles of younger patients, embarrassment of injecting in public, fear of injections, and financial constraints have been reported as important barriers among this young age group, though these influences were not explored in our study [38, 62].

Our study also showed an association between regular visits to healthcare facilities and treatment adherence. This finding is similar to other studies, including a study conducted at Felege Hiwot Referral Hospital in Ethiopia, which observed that patients who visit healthcare centres regularly were about three times more likely to adhere to insulin therapy compared to those who did not (31, 54, 55). This could be attributed to the fact that regular follow-up appointments enable patients to maintain consistent communication with their healthcare providers. This ongoing interaction between patients and healthcare providers, including pharmacists, allows for the review, reconciliation and possible adjustment of medication regimens based on patients' evolving health conditions (56, 57). Pharmacists, as part of the healthcare team, play very critical role by providing adherence counselling regarding patients' disease

management and prescribed treatments to help address issues surrounding health literacy regarding their condition (58, 59).

This study also found that individuals with type 1 diabetes exhibited lower adherence to insulin therapy when compared to those with type 2 diabetes (aOR=14.28, C.I 1.34 – 163.50, p=0.028) (Table 4). This finding contrasts with the results of studies conducted in other LMICs (25, 54) where patients with Type 2 diabetes were more likely to be non-adherent to insulin compared to those with Type 1 diabetes. This discrepancy in the findings may be attributed to the fact that the MARS-5 scale assesses independent adjustment of medicine dose as non-adherence to therapy, which may be more common among patients with Type 1 diabetes who frequently monitor their blood sugar levels and independently adjust their insulin doses accordingly. However, we cannot say this with certainty without further studies.

In addition, we found that individuals who were concurrently using oral antidiabetic medication along with insulin demonstrated greater adherence compared to those solely using insulin. This outcome differs from the results obtained in similar studies (60, 61); but are similar to others which showed low adherence among diabetes patients on insulin alone (31). This finding could be due to the same reason explained earlier where patients taking only insulin (mainly in type 1 diabetes) are prone to altering their doses which is considered as non-adherence by the MARS-5 scale.

Our study also found that participants who were employed had a better adherence level than their unemployed counterparts. This is similar to other findings where financial constraints were observed to be a determinant of adherence to insulin therapy (62). Having said this, the mode of payment for insulin had no impact on adherence to insulin therapy in our study. This needs to be investigated further especially as previous studies undertaken in Ghana and across Africa have called for greater subsidy of the prices of insulin to reduce the barrier of financial constraints to regular insulin usage (17, 19, 30, 32, 35, 36).

Finally, w[illegible]lst better adherence levels were not found to be associated with good glycemic control (i.e. fasting blood glucose of between 4 to 7 mmol/L) in our study, about three-quarters of participants with good glycemic control were adherent to insulin therapy. The lack of statistical significance of this association could be due to the relatively small sample size used for this study, and we will be looking to investigate this further in the future, considering the urgent need to treat patients with diabetes in Ghana adequately to reduce the level of complications and their associated morbidity, mortality and costs (1).

We are aware of a number of limitations with our study. Firstly, the use of fasting blood sugar levels to measure glycemic control instead of glycated hemoglobin levels may have impacted on the findings. Secondly, the type of adherence assessment tool may also have impacted on the findings, as observed above, though this tool has shown validity and reliability for several adherence studies of chronic diseases. The sample size in this study may have had an impact on the robustness of our study and the statistically significant association of our outcome variable with the many independent variables assessed. Lastly, there may be a limitation of external validity as the demography of the patients who attend the diabetes clinics were mainly university staff and students. However, we believe that the findings from our study will provide useful information to help improve the care of patients using insulin either alone or in combination with other oral therapy in Ghana and other similar settings in LMICs.

Conclusion

The level of adherence to insulin therapy at KNUST hospital was found to be sub-optimal, though this was more commonly seen among type 1 DM patients. Adherence levels were associated with patients' ages, marital status, type 2 diabetes diagnosis, regular visits to the clinic, concomitant use of oral antidiabetic medication, employment status, and the presence of co-morbidities. Some identified barriers to insulin therapy included cost, side effects, forgetfulness and a combination of cost and side effects, which need to be addressed going forward.

Recommendations

Adherence assessment and counselling by diabetes care providers on therapy-related barriers including how to deal with side effects and forgetfulness must be continually offered to patients on insulin therapy to secure their greater adherence. This must be part of the individualized and comprehensive diabetic care that target patient-specific barriers to insulin to achieve normal glycemic control, which will help to reduce diabetic complications occurrence and improve patient' quality of life. Pharmacists can play an active part in this process. Future studies to investigate the effectiveness of different interventions that can improve insulin therapy adherence in Ghana should be undertaken given the increasing burden of diabetes and its complications in the country.

Funding: This research received no external funding

Declaration of conflicts of interest: The authors have no relevant financial or non-financial interests to disclose.

Data availability statement: The data that support the findings of this study are available from the corresponding author, [IAS], upon reasonable request.

Authors' contribution: Conceptualization: IAS, MM, AAHN; methodology: IAS, MM, VB; validation: IAS, MM, AAHN; formal analysis: IS, MM, BG, VB; investigation: IAS, MM, AAHN; resources: IAS, MM; data curation: IAS, BG, VB; writing—original draft preparation: IAS, MM, AAHN, ES, JCM, BG, VB; writing—All authors; visualization: IAS, BG, VB; supervision: IAS, AAHN, VB; project administration: IAS, MM, AAHN. All authors have read and agreed to the published version of the manuscript.

References

1. Chan JCN, Lim LL, Wareham NJ, Shaw JE, Orchard TJ, Zhang P, et al. The Lancet Commission on diabetes: using data to transform diabetes care and patient lives. Lancet. 2021;396(10267):2019-82.

2. Global, regional, and national burden of diabetes from 1990 to 2021, with projections of prevalence to 2050: a systematic analysis for the Global Burden of Disease Study 2021. Lancet. 2023;402(10397):203-34.
3. Ali MK, Pearson-Stuttard J, Selvin E, Gregg EW. Interpreting global trends in type 2 diabetes complications and mortality. Diabetologia. 2022;65(1):3-13.
4. Zheng Y, Ley SH, Hu FB. Global aetiology and epidemiology of type 2 diabetes mellitus and its complications. Nat Rev Endocrinol. 2018;14(2):88-98.
5. Sun H, Saeedi P, Karuranga S, Pinkepank M, Ogurtsova K, Duncan BB, et al. IDF Diabetes Atlas: Global, regional and country-level diabetes prevalence estimates for 2021 and projections for 2045. Diabetes Res Clin Pract. 2022;183:109119.
6. IDF. International Diabetes Federation - Facts & Figures. 2023 Available at URL: https://idf.org/about-diabetes/diabetes-facts-figures/ [Accessed on 9th January, 2024]
7. Osei-Yeboah J, Owiredu W, Norgbe G, Obirikorang C, Lokpo S, Ashigbi E, et al. Physical Activity Pattern and Its Association with Glycaemic and Blood Pressure Control among People Living with Diabetes (PLWD) In The Ho Municipality, Ghana. Ethiop J Health Sci. 2019;29(1):819-30.
8. Werfalli M, Engel ME, Musekiwa A, Kengne AP, Levitt NS. The prevalence of type 2 diabetes among older people in Africa: a systematic review. Lancet Diabetes Endocrinol. 2016;4(1):72-84.
9. Godman B, Basu D, Pillay Y, Mwita JC, Rwegerera GM, Anand Paramadhas BD, et al. Review of ongoing activities and challenges to improve the care of patients with type 2 diabetes across Africa and the implications for the future. Frontiers in pharmacology. 2020;11:108.
10. WHO. World Health Organization - Ghana on the offensive against diabetes. 2023 Available at URL: https://www.afro.who.int/countries/ghana/news/ghana-offensive-against-diabetes [Accessed on 9th January, 2024]11. Gad M, Kazibwe J, Abassah-Konadu E, Amankwah I, Owusu R, Gulbi G, et al. The Epidemiological and Economic Burden of Diabetes in Ghana: A Scoping Review to Inform Health Technology Assessment. medRxiv; 2023.
12. Yorke E, Tetteh J, Boima V, Yawson AE. High BMI: an important health risk factor among older adults in Ghana. Public Health Nutr. 2021;24(14):4522-9.
13. Gatimu SM, Milimo BW, Sebastian MS. Prevalence and determinants of diabetes among older adults in Ghana. BMC Public Health. 2016;16(1):1174.
14. Tarekegne FE, Padyab M, Schröders J, Stewart Williams J. Sociodemographic and behavioral characteristics associated with self-reported diagnosed diabetes mellitus in adults aged 50+ years in Ghana and South Africa: results from the WHO-SAGE wave 1. BMJ Open Diabetes Res Care. 2018;6(1):e000449.
15. Anderson AK. Prevalence of Anemia, Overweight/Obesity, and Undiagnosed Hypertension and Diabetes among Residents of Selected Communities in Ghana. Int J Chronic Dis. 2017;2017:7836019.
16. Sarfo-Kantank O, Owusu-Dabo E, Adomako-Boateng F, Eghan BA, Dogbe JA, Bedu-Addo G. An assessment of prevalence and risk factors for hypertension and diabetes during world diabetes day celebration in Kumasi, Ghana. East African journal of public health. 2014;11:805-15.
17. Godman B, Basu D, Pillay Y, Almeida P, Mwita JC, Rwegerera GM, et al. Ongoing and planned activities to improve the management of patients with Type 1 diabetes across Africa; implications for the future. Hosp Pract (1995). 2020;48(2):51-67.
18. The L. 100 years of insulin: a technical success but an access failure. Lancet. 2021;398(10313):1777.
19. Kibirige D, Olum R, Kyazze AP, Bongomin F, Sanya RE. Availability and affordability of essential medicines and diagnostic tests for diabetes mellitus in Africa. Trop Med Int Health. 2022;27(11):942-60.
20. Mariye T, Girmay A, Birhanu T, Tasew H, Teklay G, Baraki Z, et al. Adherence to insulin therapy and associated factors among patients with diabetes mellitus in public hospitals of Central Zone of Tigray, Ethiopia, 2018: a cross-sectional study. Pan Afr Med J. 2019;33:309.
21. Alsaidan AA, Alotaibi SF, Thirunavukkarasu A, BF AL, Alharbi RH, Arnous MM, et al. Medication Adherence and Its Associated Factors among Patients with Type 2 Diabetes Mellitus Attending Primary Health Centers of Eastern Province, Saudi Arabia. Medicina (Kaunas). 2023;59(5).

22. Chefik FH, Tadesse TA, Quisido BJE, Roba AE. Adherence to insulin therapy and associated factors among type 1 and type 2 diabetic patients on follow up in Madda Walabu University Goba Referral Hospital, South East Ethiopia. PLoS One. 2022;17(6):e0269919.

23. Sendekie AK, Netere AK, Kasahun AE, Belachew EA. Medication adherence and its impact on glycemic control in type 2 diabetes mellitus patients with comorbidity: A multicenter cross-sectional study in Northwest Ethiopia. PLoS One. 2022;17(9):e0274971.

24. Dabas H, Sarin J, Madhu SV. Insulin Adherence in Adolescents with Type 1 Diabetes Mellitus. Indian J Endocrinol Metab. 2023;27(5):394-7.

25. Farsaei S, Radfar M, Heydari Z, Abbasi F, Qorbani M. Insulin adherence in patients with diabetes: risk factors for injection omission. Prim Care Diabetes. 2014;8(4):338-45.

26. Skriver LKL, Nielsen MW, Walther S, Nørlev JD, Hangaard S. Factors associated with adherence or nonadherence to insulin therapy among adults with type 2 diabetes mellitus: A scoping review. J Diabetes Complications. 2023;37(10):108596.

27. Nieuwlaat R, Wilczynski N, Navarro T, Hobson N, Jeffery R, Keepanasseril A, et al. Interventions for enhancing medication adherence. Cochrane Database Syst Rev. 2014;2014(11):Cd000011.

28. Kvarnström K, Westerholm A, Airaksinen M, Liira H. Factors Contributing to Medication Adherence in Patients with a Chronic Condition: A Scoping Review of Qualitative Research. Pharmaceutics. 2021;13(7).

29. Stolpe S, Kroes MA, Webb N, Wisniewski T. A Systematic Review of Insulin Adherence Measures in Patients with Diabetes. J Manag Care Spec Pharm. 2016;22(11):1224-46.

30. Awuni Prosper Mandela A. Non Adherence to Diabetic Medication Among Diabetic Patients, a Case Study of Dormaa Hospital Ghana. Science Journal of Public Health. 2017;5(2):88-97.

31. Rwegerera GM, Masaka A, Pina-Rivera Y, Moshomo T, Gaenamong M, Godman B, et al. Determinants of glycemic control among diabetes mellitus patients in a tertiary clinic in Gaborone, Botswana: findings and implications. Hosp Pract (1995). 2019;47(1):34-41.

32. Ewen M, Joosse HJ, Beran D, Laing R. Insulin prices, availability and affordability in 13 low-income and middle-income countries. BMJ Glob Health. 2019;4(3):e001410.

33. Godman B, Haque M, Leong T, Allocati E, Kumar S, Islam S, et al. The Current Situation Regarding Long-Acting Insulin Analogues Including Biosimilars Among African, Asian, European, and South American Countries; Findings and Implications for the Future. Front Public Health. 2021;9:671961.

34. EML. Essential Medicine List, Ministry of Health, Ghana National Drug Programme, 7th Edition. 2017. Accessed from: https://www.moh.gov.gh/wp-content/uploads/2020/07/GHANA-EML-2017.pdf o on the 9th January, 202435. Godman B LT, Abubakar AR, Kurdi A, Kalemeera F, Rwegerera GM, et al. Availability and Use of Long-Acting Insulin Analogues Including Their Biosimilars across Africa: Findings and Implications. Intern Med. 2021;11(4):1 - 17.

36. Tagoe ET, Nonvignon J, van Der Meer R, Megiddo I, Godman B. Challenges to the delivery of clinical diabetes services in Ghana created by the COVID-19 pandemic. J Health Serv Res Policy. 2023;28(1):58-65.

37. Atinga RA, Yarney L, Gavu NM. Factors influencing long-term medication non-adherence among diabetes and hypertensive patients in Ghana: A qualitative investigation. PLoS One. 2018;13(3):e0193995.

38. Zhuo Y, Pan Y, Lin K, Yin G, Wu Y, Xu J, et al. Effectiveness of clinical pharmacist-led smartphone application on medication adherence, insulin injection technique and glycemic control for women with gestational diabetes receiving multiple daily insulin injection: A randomized clinical trial. Prim Care Diabetes. 2022;16(2):264-70.

39. Xin C, Xia Z, Jiang C, Lin M, Li G. Effect of pharmaceutical care on medication adherence of patients newly prescribed insulin therapy: a randomized controlled study. Patient Prefer Adherence. 2015;9:797-802.

40. Selvadurai S, Cheah KY, Ching MW, Kamaruddin H, Lee XY, Ngajidin RM, et al. Impact of pharmacist insulin injection re-education on glycemic control among type II diabetic patients in primary health clinics. Saudi Pharm J. 2021;29(7):670-6.

41. Afaya RA, Bam V, Azongo TB, Afaya A, Kusi-Amponsah A, Ajusiyine JM, et al. Medication adherence and self-care behaviours among patients with type 2 diabetes mellitus in Ghana. PLoS One. 2020;15(8):e0237710.
42. Opoku R, Ackon SK, Kumah E, Botchwey CO, Appiah NE, Korsah S, et al. Self-care behaviors and associated factors among individuals with type 2 diabetes in Ghana: a systematic review. BMC Endocr Disord. 2023;23(1):256.
43. Bruce SP, Acheampong F, Kretchy I. Adherence to oral anti-diabetic drugs among patients attending a Ghanaian teaching hospital. Pharm Pract (Granada). 2015;13(1):533.
44. Sefah IA, Okotah A, Afriyie DK, Amponsah SK. Adherence to Oral Hypoglycemic Drugs among Type 2 Diabetic Patients in a Resource-Poor Setting. Int J Appl Basic Med Res. 2020;10(2):102-9.
45. Emily Milla-Amekor STOISEAE. Factors Influencing Adherence to Anti-Diabetes Medication Among Diabetes In Accra. CEMJP. 2023;31(1):690-705.
46. Khoiry QA, Alfian SD, van Boven JFM, Abdulah R. Self-reported medication adherence instruments and their applicability in low-middle income countries: a scoping review. Front Public Health. 2023;11:1104510.
47. Chan AHY, Horne R, Hankins M, Chisari C. The Medication Adherence Report Scale: A measurement tool for eliciting patients' reports of nonadherence. Br J Clin Pharmacol. 2020;86(7):1281-8.
48. Konstantinou P, Kasinopoulos O, Karashiali C, Georgiou G, Panayides A, Papageorgiou A, et al. A Scoping Review of Methods Used to Assess Medication Adherence in Patients with Chronic Conditions. Ann Behav Med. 2022;56(12):1201-17.
49. Wibowo M, Yasin NM, Kristina SA, Prabandari YS. Exploring of Determinants Factors of Anti-Diabetic Medication Adherence in Several Regions of Asia - A Systematic Review. Patient Prefer Adherence. 2022;16:197-215.
50. Moeed A, Najeeb H, Saleem A, Asghar MS, Rafi HM, Khattak AK, et al. Willingness and Perceptions Regarding COVID-19 Vaccine Booster Dose in Pakistani Vaccinated Population: A Cross-Sectional Survey. Frontiers in Public Health. 2022;10.
51. Vishwanathan K, Patel GM, Patel DJ. Impact and perception about distant online medical education (tele-education) on the educational environment during the COVID-19 pandemic: Experiences of medical undergraduate students from India. Journal of Family Medicine and Primary Care. 2021;10(6):2216-24.
52. Monami M, Marchionni N, Mannucci E. Long-acting insulin analogues versus NPH human insulin in type 2 diabetes: a meta-analysis. Diabetes research and clinical practice. 2008;81(2):184-9.
53. Shafie AA, Ng CH, Tan YP, Chaiyakunapruk N. Systematic review of the cost effectiveness of insulin analogues in type 1 and type 2 diabetes mellitus. Pharmacoeconomics. 2017;35:141-62.
54. Tewabe T, Kindie S. Level of insulin adherence among diabetes mellitus patients in Felege Hiwot Referral Hospital, Bahir Dar, Northwest Ethiopia, 2017: a cross-sectional study. BMC Res Notes. 2018;11(1):295.
55. Riaz M, Basit A, Fawwad A, Yakoob Ahmedani M, Ali Rizvi Z. Factors associated with non-adherence to insulin in patients with type 1 diabetes. Pak J Med Sci. 2014;30(2):233-9.
56. Paulino E, Thomas D, Lee SWH, Cooper JC. Dispensing process, medication reconciliation, patient counseling, and medication adherence. Clinical Pharmacy Education, Practice and Research: Elsevier; 2019. p. 109-20.
57. Ryan TP, Morrison RD, Sutherland JJ, Milne SB, Ryan KA, Daniels JS, et al. Medication adherence, medical record accuracy, and medication exposure in real-world patients using comprehensive medication monitoring. PLoS One. 2017;12(9):e0185471.
58. Moosa A, Bezuidenhout S, Meyer JC, Godman B. Knowledge regarding medicines management of type 2 diabetes amongst patients attending a Community Health Centre in South Africa. Journal of Pharmaceutical Health Services Research. 2019;10(1):13-28.

59. Geurts MM, Talsma J, Brouwers JR, de Gier JJ. Medication review and reconciliation with cooperation between pharmacist and general practitioner and the benefit for the patient: a systematic review. British journal of clinical pharmacology. 2012;74(1):16-33.
60. Ayele AA, Tegegn HG, Ayele TA, Ayalew MB. Medication regimen complexity and its impact on medication adherence and glycemic control among patients with type 2 diabetes mellitus in an Ethiopian general hospital. BMJ Open Diabetes Res Care. 2019;7(1):e000685.
61. Rwegerera GM. Adherence to anti-diabetic drugs among patients with Type 2 diabetes mellitus at Muhimbili National Hospital, Dar es Salaam, Tanzania- A cross-sectional study. Pan Afr Med J. 2014;17:252.
62. Sharma T, Kalra J, Dhasmana D, Basera H. Poor adherence to treatment: A major challenge in diabetes. Journal, Indian Academy of Clinical Medicine. 2014;15.

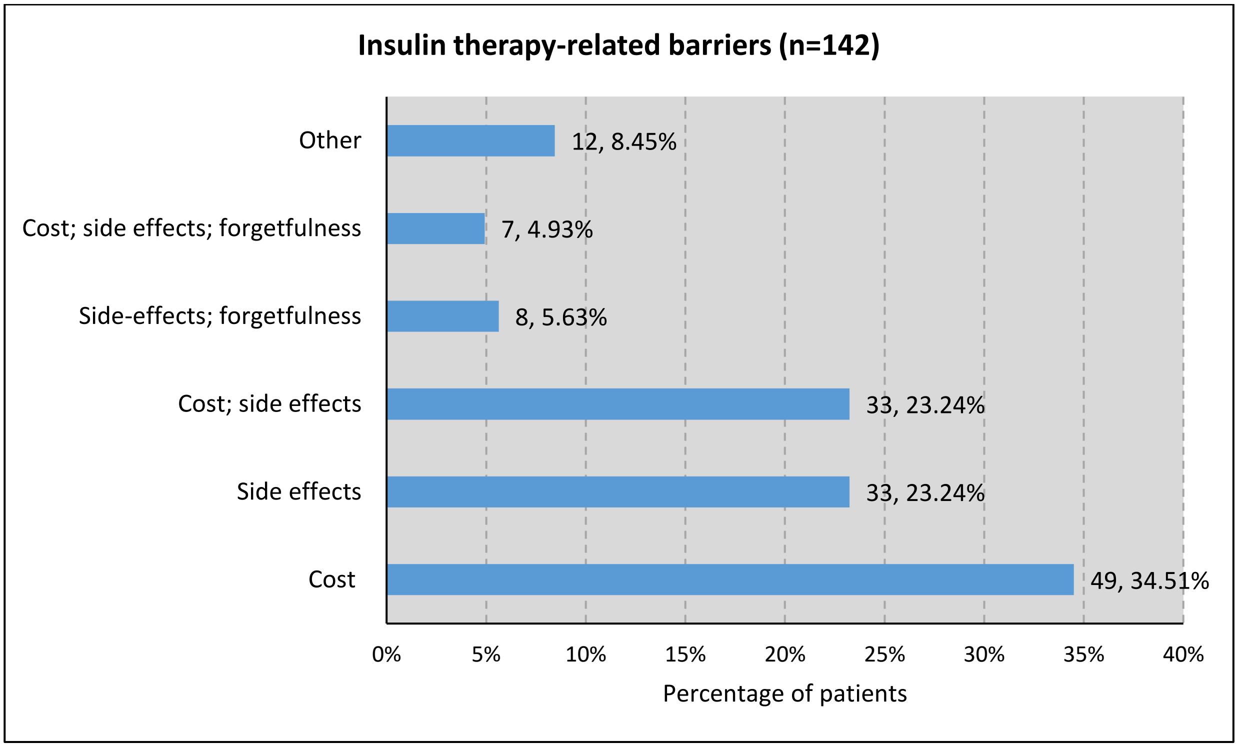

Figure 1: Common insulin therapy-related barriers patients encountered (n=142)
