# Supplementary material for: Insulin therapy adherence and its associated factors among diabetic patients in a Ghanaian primary care hospital
Source: PLoS One. 2025 Jan 24;20(1):e0312094. doi: 10.1371/journal.pone.0312094 (PMC11760006; doi:10.1371/journal.pone.0312094)
Supplement: Attachment — Submitted filename: Response to Reviewers.docx [file pone.0312094.s002.docx]

**REVIEWERS’ COMMENTS AND AUTHORS’ RESPONSES**

**Insulin Therapy Adherence and its Associated Factors among Diabetic Patients in a Ghanaian Primary Care Hospital**

Reviewer #1: Dear Authors,
Thank you for your submission. Please note the following comments and suggestions

Abstract- Background - please introduce a sentence to provide an insight into the problem before stating the last sentence on the purpose of the study.

AUTHOR RESPONSE AND ACTION TAKEN: Thank you for this. We have now added a statement defining the problem to the background section of the abstract

Introduction
L93, it is important to insert the year of reference instead of "currently".
Is there any reason for prevalent in some specific regions etc?.
There is a need to strengthen the novelty of the study.

AUTHOR RESPONSE AND ACTION TAKEN: Thank you for this suggestion. We have now added the year of reference and additional details to strengthen the novelty and relevance of the study.

It is suggested that authors conduct a literature review on insulin therapy adherence. Please insert a section on that.

AUTHOR RESPONSE AND ACTION TAKEN: Thank you for this. We have now added more details to show literature review conducted on the subject matter (line 102 to 106).

Method
Does the researchers have an idea of the study total population size before the adopted sample size?.

AUTHOR RESPONSE AND ACTION TAKEN: We based the calculation of our sample size on the average monthly outpatient attendance of 200 giving us a total of 600 patients to be expected at the clinic over the 3 months’ duration of data collection. We have now updated that section of the method to bring clarity to our estimation (line 152 to 155)

Discussion
There is a need to explore through the results, the impact of lifestyle and status on the study focus. Also, in the introduction, Authors mentioned the disparate records from the different regions in Ghana, does the study explore this aspect?
More can be achieved from the collected data.

AUTHOR RESPONSE AND ACTION TAKEN: Thank you for this comment. Our study was done in just one hospital which provides service to patients living just around the hospital and so we could not explore the different prevalence levels at different regions and settings. We have indicated this as a limitation to our study (line303 to 305).

Please create a section for the implication of the study.

AUTHOR RESPONSE AND ACTION TAKEN:

Conclusion
Kindly review and enhance this to provide a robust Conclusion to the study.
AUTHOR RESPONSE AND ACTION TAKEN:

Please review the manuscript for grammatical errors.

AUTHOR RESPONSE AND ACTION TAKEN: Thank you for this. We have now updated the manuscript by correcting identified grammatical errors.

Reviewer #2: - The authors provide a good argument for the survey. The review of the literature is adequate, however I disagree that Insulin therapy is solely responsible for the outcomes in patients with diabetes mellitus (line 106 -108).
- The aim of the study is clear
- The target population is well defined
- The inclusion criteria is well defined

AUTHOR RESPONSE AND ACTION TAKEN: Thank you for your comments.

- The authors provide a clear description of the study processes, the data collection instrument and the statistical instruments used in the data analysis.

AUTHOR RESPONSE AND ACTION TAKEN: Thank you for this.

- The results are presented clearly in accurate tables

AUTHOR RESPONSE AND ACTION TAKEN: We are very grateful for this comment.

- The discussion is adequate but the authors should please look at the reviewer comments made on lines 263, 293-296, 303 - 311. The authors show a clear understanding of the limitations of the study method and processes

AUTHOR RESPONSE AND ACTION TAKEN: Thank you for this. We have now updated the manuscript based on the comments made concerning those portions of the paper to more details on the relevance of our findings and the limitations in our study

- The concluding statement is adequate. The recommendations made are reasonable based on the findings.

AUTHOR RESPONSE AND ACTION TAKEN: We are very grateful for this.

- The authors should please make minor corrections to the references high lighted. 10,11 and 34,35.

AUTHOR RESPONSE AND ACTION TAKEN: We have now corrected the errors in the references identified. Thank you.
