# Supplementary material for: Insulin therapy adherence and its associated factors among diabetic patients in a Ghanaian primary care hospital
Source: PLoS One. 2025 Jan 24;20(1):e0312094. doi: 10.1371/journal.pone.0312094 (PMC11760006; doi:10.1371/journal.pone.0312094)
Supplement: Attachment — Submitted filename: Response to reviewers.docx [file pone.0312094.s003.docx]

**AUTHORS RESPONSE TO REVIEWER COMMENTS**

**REVIEWER 2**

Comment: The authors have given a clear description of the processes for the sampling and administering of the data collection tool.

Authors response: We are grateful for this comment.

Comment: The data analysis used had adequate statistical instruments to test the associations between dependent and independent variables.

Authors response: We are grateful for this comment.

Comment: The presentation of the results are clear and precise. The tables are accurate

Authors response: Thank you for this comment.

Comment: The authors need to review the document for minor errors such as this - level of adherence OR adherence level...

Authors response: Thank you for this correction. The document has now been updated by removing the error “level of”.

Comment: This argument is unclear as the authors state in the opening statement to the paragraph that the employed were less likely to be non-adherent. The mode of payment may be an entirely different argument. What did the authors mean by 'mode of payment' in the MARS-5 tool that was used?

Authors response: Thank you for comment. The mode of payment was not part of the MARS-5 question but was added to assess how cash payment and/ or insurance has an influence on adherence. We believe that cash payment for insulin and employment status are all economic factors that may influence adherence level. We have added extra details in our manuscript to explain this (Lines 308-309).

Comment: Another limitation of this study is the fact that authors did not look at the subjective aspects of control that may limit the level of control in patients. All the arguments are solely focused on adherence to insulin therapy excluding the other aspects of diabetes care that could serve as confounders to the findings especially relating to control of serum glucose levels.

Authors response: Thank you for this input. We have now included this as an important confounding factor that was not assessed (Line 325-327).

Comment The concluding statement is adequate as it focuses on the findings related to the aim of the study.

Authors response: We are grateful for this comment.

Comment: Please review this reference for the appropriate Vancouver style. It also looks like two references put together in error.

Authors response: Thank you for this correction. The errors with references 10 and 11 are now corrected (Lines 387-390).

Comments: Authors should please correct the errors in the reference list.

Authors’ response: Thank you for this. The references have been updated to Vancouver style.

Comment Please make the necessary corrections to this reference. Please reference Vancouver style.

Authors response: Thank you for this correction. The errors with reference numbers 34 and 35 are now corrected (Line 450 – 452).

**REVIEWER 3**

Comment: There were omissions in line 51- it should be Kwame Nkrumah University of Technology, this is repeated in line 140.

Authors response: Thank you for this. Corrections have now been made to this update. (Lines 36 and 127)

Comment: In line 46, the authors should be clear on which patients are place on insulin by stating all types is not clear.

Authors response: Thank you for this. The error is now corrected in line 147.

Comment: The sampling utilized in the study was convenience sampling, author was silent on how this was done at the facility and how many days were used to recruit participants for the study.

Authors response: We have now on how patients were recruited using the convenience sampling technique (line 146-147). The interview duration is captured in the manuscript at line 164-165

Comment: The abstract of the study needs review especially on all types of diabetes.

Authors response: Thank you for this correction. We have updated the manuscript (Line 30)

Comment: The inclusion criteria included persons older than 18 years however, in the results section, the age range was between 16-20, author should address the difference in age.

Authors response: Thank you for this. We have now corrected the age range to 18-20 years.

Comment: It is not clear if all the type 2 diabetes were on the combine medication of insulin and oral glycemic agents.

Authors response: This is not so in our study. It is worthy to note that a significant number of the patients were on oral therapy (n=156) which is closer to the number of type 2 DM (n=159) in our study.

Comment: The references had a different font.

Authors response: Thank you for this. We have now formatted the references.
